# Risk Factors of At-Risk/Problem Gambling Among Young Adult Spanish Students

**DOI:** 10.1007/s10935-024-00814-x

**Published:** 2024-11-21

**Authors:** A. Krotter, R. Secades-Villa, C. Iza-Fernández, A. González-Roz

**Affiliations:** Addictive Behaviors Research Group (GCA), Department of Psychology, Faculty of Psychology, University of Oviedo, Plaza Feijoo S/N, 33003 Oviedo, Spain

**Keywords:** Prevention, Problem gambling, Risk factors, Young adults

## Abstract

The gambling industry in Spain has experienced sustained growth in recent years. This has raised concerns about its potential impact, particularly during emerging adulthood, which is a high-risk period for the development of problematic gambling behaviors. The aim of this study was to estimate the prevalence of problem gambling in young Spanish adults and to examine individual factors associated with at-risk or problem gambling. The participants were 2762 young adults studying at university and vocational schools (64.16% women, *M*_age_ = 19.47; *SD* = 1.64) recruited in three Spanish autonomous communities. Individuals completed an e-assessment including sociodemographic variables, gambling-related characteristics, and the Problematic Gambling Severity Index. A total of 3.51%, 2.14%, and 0.51% were low risk, moderate risk, and problem gamblers, respectively. Predictors of at-risk/problem gambling were early gambling onset (*OR* 0.790; 95% *CI* 0.672, .929), mixed versus offline modality gambling access (*OR* 2.216; 95% *CI* 1.396, 3.516), and having gambled in the previous year in a casino (*OR* 1.910; 95% *CI* 1.194, 3.056), sports betting (*OR* 1.597; 95% *CI* 1.003, 2.542), or electronic gaming machines (*OR* 2.111; 95% *CI* 1.064, 4.189). Gambling is ubiquitous in the general population and a high proportion of young adults present low and moderate risk of problem gambling. Addressing gambling is a public health challenge and policies to regulate it should be targeted at delaying the age of onset and restricting access to online gambling.

**Supplementary Information:**

The online version contains supplementary material available at 10.1007/s10935-024-00814-x.

## Introduction

Gambling is a common activity all over the world, including Spain (Chóliz et al., 2021), where 58.1% of adults gambled during 2022 (National Plan on Drugs, 2022). In the same year, 24% of Spaniards aged 15–24 years old gambled offline and 6.5% gambled online (National Plan on Drugs, 2022). Although gambling is often regarded as a leisure activity, it can result in problematic behaviors that impact an individual’s work, family, and social life (Tulloch et al., 2022). The prevalence of problem gambling worldwide has been estimated between 1.29 and 1.41% among adults (Gabellini et al., 2023; Tran et al., 2024).

The global gambling industry continues to demonstrate consistent and sustained expansion (Guillou-Landreat et al., 2021). In Spain specifically, the gambling market has seen dramatic growth over the last ten years, both financially (Spanish Directorate General for the Regulation of Gambling, 2022) and in the amount of gambling opportunities on offer (Pérez et al., 2022). Increased investment in advertising, promotions, and sponsorships has resulted in a shift in Spaniards’ gambling behavior, as evidenced by the progressive increase in the number of new online gambling accounts from 2013 to 2023 (García-Pérez et al., 2024). Moreover, additional research in Spain has demonstrated the impact of the proliferation of on-site betting shops close to secondary schools, particularly in less advantaged areas (Espadafor & Martínez, 2021), as well as an increased use of tipsters for online betting among young adults (Gonzálvez-Vallés et al., 2021). The expansion of gambling opportunities has increased public health concerns about gambling and its harmful consequences (Dickson-Gillespie et al., 2008) which has resulted in calls for further investigation into the gambling patterns of the Spanish population.

The period of emerging adulthood, which encompasses the ages of 18–25, is a vulnerable phase of development. During this period, individuals are no longer subject to the monitoring that is characteristic of adolescence, but have not yet assumed the responsibilities associated with adulthood (Sussman & Arnett, 2014). This results in numerous changes in education, work, and interpersonal interactions, which may precipitate risky behaviors and increase the likelihood of developing addiction-related problems in general (Stone et al., 2012) and gambling-related problems in particular (Allami et al., 2021).

Despite the expansion of the gambling industry in Spain and the elevated risk for problem gambling behaviors during emerging adulthood, research on the prevalence and risk factors of problem gambling in young Spanish adults is scarce. One prior study found a rate of problem gambling of 1.04% in Spanish 18- to 25-year-olds (Chóliz et al., 2021). Another study classified 2.4% of university students (*M*_age_ = 20.57; *SD* = 2.37) as possible problem gamblers (López-Del-Hoyo et al., 2022). Given that the participants in these previous studies were recruited in 2016 (Chóliz et al., 2021), or were attending a single Spanish university (López-Del-Hoyo et al., 2022), further research is necessary to ascertain the prevalence of problem gambling among young Spanish adults. The finding that the prevalence of problem gambling is higher among Spanish 15- to 25-year-olds than in their counterparts in the United States, South Korea, and Finland (Oksanen et al., 2021) also underscores the importance of continued monitoring of gambling behaviors in this vulnerable demographic.

To date, the literature on problem gambling among young adults has identified several risk factors, such as being male (Edgerton et al., 2015, 2019; Noel et al., 2023), being an older young adult (Noel et al., 2023; Scholes‐Balog et al., 2014), gambling online (Tomei et al., 2022) or in a mixed mode (i.e., both online or offline) (Marmet et al., 2021), sports betting (Noel et al., 2023), maladaptive coping strategies (Edgerton et al., 2015), impulsivity (Bilevicius et al., 2020; Edgerton et al., 2015), or substance use (Bilevicius et al., 2020; Jang et al., 2019). It is notable that none of the above studies were conducted in Spain. Surprisingly, despite several studies examining the risk factors associated with problem gambling among Spanish adolescents (González-Roz et al., 2017; Pérez-Albéniz et al., 2021) or adults (Díaz & Pérez, 2021; Secades-Villa et al., 2023), very few studies have evaluated this issue among emerging adults specifically. To our knowledge, the only available study conducted with a Spanish sample (Oksanen et al., 2021) identified several risk factors associated with problem gambling (e.g., impulsivity, perceived social support, online casino gambling). However, the study sample also included adolescents (specifically individuals aged from 15 to 25 years old), which makes it difficult to extrapolate the findings to the population of young adults, and the sample size was relatively small (1212 individuals).

Considering this background, especially the expansion of the gambling industry in Spain and the dearth of studies analyzing the risk factors of at-risk/problem gambling in the Spanish population aged 18–25 years of age, further research is required in this area. A more nuanced understanding of the risk factors for problem gambling among young adults in Spain will be instrumental in the development of prompt detection methodologies and the implementation of effective prevention initiatives. Thus, the primary aim of this study was to estimate the prevalence of at-risk/problem gambling among young Spanish adults and to identify sociodemographic and gambling-related risk factors for at-risk/problem gambling in this population.

## Method

### Participants

The participants were young adult undergraduates studying at university or vocational schools from three autonomous communities in Spain (Aragon, Principality of Asturias, and The Balearic Islands) and were recruited by non-probability sampling via print (flyers and posters) and mass media advertising (radio, TV, Instagram, and Twitter). Flyers and posters were posted on bulletin boards in universities and vocational schools to inform people about a study aimed at evaluating addictive behaviors among young people, including alcohol, tobacco, cannabis, gaming and gambling. Furthermore, the coordinators of randomly selected university degree courses were contacted, told about the project, and asked to collaborate and allow access for the assessments to be carried out during teaching hours.

The recruitment was conducted during September–November 2021, meaning it is useful to review the health situation in the country at that time with respect to the coronavirus pandemic. During this period, stay-at-home orders due to COVID-19 were not in place and classes at the university were conducted face-to-face (i.e., not online). Stores, bars and other businesses, such as indoor recreational activity facilities, were open with some limitations (e.g., use of masks), and there were no specific restrictions on gambling. Therefore, based on the situation in Spain, we consider the study timeframe to be “post-pandemic”, despite the World Health Organization declaring the end of the COVID-19 pandemic in May 2023 worldwide. The eligibility criteria were: (1) being aged 18–25, and (2) willingness to participate in future assessments (specifically, 3 times in total every six months).

The final sample comprised 2762 participants with a mean age of 19.47 (*SD* = 1.64) years, who were mostly women (64.16%). The initial assessment was completed by 2980 individuals. Applying the eligibility criteria, 121 were excluded due to being over 25 years old, and an additional 75 cases were discarded because they responded to the survey twice. In addition, 22 subjects failed attentional control checks. To verify sufficient effort and attention to the task, there were four attentional control items (e.g., for this question choose “true”) included within the battery assessment. To be included in the analyses, participants had to answer at least half of the control checks correctly.

### Procedure

Participants gave their written informed consent before the study started, and completed an assessment (https://www.metajovenes.es) lasting approximately 45 min, either using their own devices or tablets from the research project (Lenovo® Tab M7). Participation was online or in person (approximately half of the participants completed the assessments online). To encourage participation, participants were entered into raffles with prizes of €50 vouchers. The study protocol #191CER21) was approved by the ethics committee from the university that received the funding.

### Measures

Sociodemographic measures included sex, age, employment status, amount of money available per week (either earned on their own or received from parents or other relatives), and family financial situation—which was measured via subjective financial status (i.e., not good enough to be able to pay the bills, good enough to pay the bills without additional expenses, or good enough to pay the bills and additional expenses).

Gambling behavior was assessed by asking the participants if they had gambled within the previous year, as well as the age of gambling onset. Gambling access modality was either exclusively offline, exclusively online, or mixed. Past-year gambling activities were assessed considering the venues noted previously, and included: bingo, poker, casino games, sports betting, lotteries, scratch-cards, slot-machines, other electronic gaming machines (EGMs; e.g., virtual roulette), trading (i.e., the stock-market or cryptocurrency-market), and private bets with friends (e.g., at home). Gamblers were categorized—based on the type of games they had engaged in within the previous 12 months—as strategic gamblers (i.e., having gambled exclusively on activities which emphasize individual skills, such as poker or sports betting), non-strategic gamblers (i.e., having gambled solely on games of chance, such as bingo or slot-machines), or mixed gamblers (i.e., having gambled on both strategic and non-strategic games) (Moragas et al., 2015). Finally, participants were asked about perceived gambling accessibility in their community (i.e., not available, or available).

Gambling severity was assessed through the Problem Gambling Severity Index (PGSI) (López-González et al., 2018). The PGSI comprises nine items on a 4-point Likert scale from 0 (*never*) to 3 (*almost always*), with a total score ranging from 0 to 27. Four groups were identified based on the PGSI total score: non-problem gamblers (PGSI = 0), low-risk gamblers (PGSI = 1–2), moderate-risk gamblers (PGSI = 3–7), and problem gamblers (PGSI ≥ 8). This scale showed excellent psychometric properties in a sample of Spanish adults, with an internal consistency of 0.97 (López-González et al., 2018). The PGSI also showed a good internal consistency in the present study (α = 0.818).

### Statistical Analyses

The pipeline statistical approach was as follows: first, we conducted a set of univariate descriptive statistics (i.e., means and frequencies) to characterize the study sample in terms of sociodemographic and gambling-related characteristics. The data distribution was checked for normality to determine the suitability of parametric or non-parametric tests.

To ensure a large enough sample for the analysis, we considered two groups of gambling severity categorized from the PGSI scores: non-problem gamblers (i.e., PGSI = 0) and at-risk/problem gamblers (i.e., PGSI ≥ 1), as in prior studies (Martínez-Loredo et al., 2019; Potenza et al., 2011). Differences between gambling severity groups were analyzed using the Mann–Whitney *U* test for continuous variables (e.g., money available or gambling age onset) and chi-square for categorical variables (e.g., sex or gambling access modality). Effect sizes were calculated by *r* = *Z / √n*, and Cramer’s *V.* Results from this comparison analyses are provided for descriptive purposes.

Finally, a backward stepwise logistic regression was carried out, using gambling severity group (at-risk/problem gambling versus non-problem gambling) as the dependent variable. Independent variables that showed significant differences between gambling severity groups in the bivariate analyses were added to the regression, specifically: sex, age of gambling onset, gambling access modality (i.e., offline, online, or mixed modality), type of gambler (strategic, non-strategic, or mixed gambler), number of gambling activities online, number of gambling activities offline, and past-year gambling in casino, sports betting, lotteries, scratch-tickets, EGM, trading, and private bets with friends. To ensure sufficient power, a two-tailed post hoc power analysis was conducted using an α error probability of 0.05 in a sample size of 451. This analysis yielded a 100% power to detect significant differences with an OR of 2.33. Confidence levels were set 95%, and statistical analyses were conducted using the SPSS package (V. 20, Inc, Chicago, IL).

## Results

### Participants’ Characteristics

Table 1 presents the sociodemographic and gambling-related characteristics of the participants who had engaged in gambling activities within the previous year (*n* = 451). The non-gambling participants (*n* = 2311) were predominantly female (68.80%; 1590/2311) with a mean age of 19.48 years old (*SD* = 1.67), and 17.14% (396/2311) were employed. The mean amount of money available per week was €56.65 (*SD* = €126), and the most frequently reported family financial situation was “good enough to pay the bills without additional expenses” (56.38%, 1303/2311).

**Table 1:** Sociodemographic and gambling-related characteristics of individuals with and without risk/problem gambling

	Non-problem gamblers (*n* = 281)	At-risk/problem gamblers (*n* = 170)	*p*	*Effect size*
*Sociodemographic*				
Male (n/%)	152 (54.09)	117 (68.82)	**0.003**	0.146
Age^a^	19.39 (1.52)	19.39 (1.45)	0.775	–
Employed (*n*/%)	54 (19.22)	26 (15.29)	0.352	–
Money available per week ^a^	77.04 (218.74)	73.39 (197.59)	0.942	–
Family financial situation (*n*/%)			0.218	–
Not good enough to pay the bills	14 (4.98)	6 (3.53)		
Good enough to pay the bills without additional expenses	142 (50.53)	100 (58.82)		
Good enough to pay the bills and additional expenses	125 (44.48)	64 (37.65)		
*Gambling behavior*				
Age at onset^a^	18.11 (1.68)	17.60 (1.75)	** < 0.001**	0.209
Access modality (*n*/%) ^b^			** < 0.001**	0.249
Offline	181 (64.87) _d_	67 (39.88) _e_		
Online	11 (3.94) _d_	7 (4.17) _d_		
Mixed	87 (31.18) _d_	94 (55.95) _e_		
Type of gambler (*n*/%) ^c^			**0.011**	0.143
Strategic	92 (33.45) _d_	48 (28.92) _d_		
Non-strategic	44 (16) _d_	13 (7.83) _e_		
Mixed	139 (50.55) _d_	105 (63.25) _e_		
Total No of gambling activities offline^a^	2.44 (1.59)	3.21 (1.96)	** < 0.001**	0.198
Total no gambling activities online^a^	0.60 (1.08)	1.29 (1.66)	** < 0.001**	0.264
Gambling availability (*n*/%)			0.065	–
Not available	47 (16.73)	17 (10)		
Available	234 (83.27)	153 (90)		
*Gambling activities offline or online*				
*(12-months, n/%)*				
Bingo	100 (35.59)	60 (35.29)	1	–
Poker	48 (17.08)	32 (18.82)	0.732	–
Casino	171 (60.85)	123 (72.35)	**0.017**	0.117
Sports betting	105 (37.37)	103 (60.59)	** < 0.001**	0.226
Lotteries	94 (33.45)	74 (43.53)	**0.041**	0.101
Scratch-cards	77 (27.40)	63 (37.06)	**0.041**	0.101
Slot-machine	39 (13.88)	34 (20)	**0.114**	–
Other EGM	21 (7.47)	31 (18.24)	**0.001**	0.163
Trading	32 (11.39)	41 (24.12)	**0.001**	0.167
Private bets with friends	81 (28.83)	69 (40.59)	**0.014**	0.121

Statistically significant differences are highlighted in bold

EGM = electronic gaming machine. Groups with the same subscript did not differ significantly from each other

^a^*M* (*SD*); ^b^ *n* = 4 missing data; ^c^*n* = 10 missing data

### Problem Gambling Prevalence and Gambling Related Characteristics

Around one sixth of the participants (16.33%; 451/2762) reported having gambled in the previous year. Considering the total sample, 10.17% of the subjects (281/2762) were classified as non-problem gamblers, 3.51% (97/2762) as low-risk gamblers, 2.14% (59/2762) as moderate-risk gamblers, and 0.51% (14/2762) as problem gamblers. Among those who had gambled in the previous year, the prevalence rates were: non-problem gamblers, 62.31% (281/451); low-risk, 21.51% (97/451); moderate-risk, 13.08% (59/451); and problem gamblers, 3.10% (14/451).

The average age of gambling onset was 17.92 (*SD* = 1.73). Most participants gambled exclusively offline during the previous year (55.48%), followed by mixed modality (40.49%), and a minority exclusively online (4.03%). A total of 55.33% were classified as mixed-gamblers, 31.75% as strategic gamblers, and the remaining 12.93% as non-strategic. Individuals’ specific gambling during the past year were: casino (65.19%), sports betting (46.12%), lotteries (37.25%), bingo (35.47%), private bets with friends (33.26%), scratch-cards (31.04%), poker (17.74%), slot-machines (16.19%), trading (16.19%), and EGMs (11.53%).

### Differences in Sociodemographic and Gambling-Related Characteristics Between At-Risk/Problem Gamblers and Non-Problem Gamblers

Table 1 shows that men were significantly more likely than women to be at-risk/problem gamblers (*p* = 0.003). Compared to non-problem gamblers, at-risk/problem gamblers demonstrated earlier gambling onset (*p* < 0.001), gambled more in mixed modality (*p* < 0.001), reported both strategic and non-strategic gambling engagement more frequently (*p* = 0.011), and reported gambling in more offline (*p* < 0.001) and online (*p* < 0.001) activities. At-risk/problem gamblers gambled more frequently than non-problem gamblers in the following activities (either online or offline): casino (*p* = 0.017), sports betting (*p* < 0.001), lotteries (*p* = 0.041), scratch-cards (*p* = 0.041), EGMs (*p* = 0.001), trading (*p* = 0.001), and private bets with friends (*p* = 0.014). Offline modality and non-strategic gambling were more frequently reported by non-problem gamblers than at-risk/problem gamblers (all *p* values < 0.014).

### Predictors of At-Risk/Problem Gambling

Significant predictors of at-risk/problem gambling were: earlier gambling onset [*B* = -0.236; *OR* 0.790 (95% *CI* 0.672, 0.929)]; mixed modality gambling versus exclusively offline [*B* = 0.796; *OR* 2.216 (95% *CI* 1.396, 3.516)]; past-year casino [*B* = 0.647; *OR* 1.910 (95% *CI* 1.194, 3.056)]; past-year sports betting [*B* = 0.468; *OR* 1.597 (95% *CI* 1.003, 2.542)]; and past-year EGMs [*B* = 0.747; *OR* 2.111 (95% *CI* 1.064, 4.189)]. The final model explained 17.7% of the variance (see Table 2). The complete regression model is presented in Table S1.

**Table 2:** Results from logistic regression of sociodemographic and gambling-related characteristics for respondents with at-risk/problem gambling

	*R*^2^ Nagelkerke	*B*	*SE*	*p*	*OR* [95% *CI*]
Last step (model 8)	0.177				
Age at gambling onset		− 0.236	0.082	**0.004**	0.790 [0.672, 0.929]
Exclusively online access^a^		0.685	.594	0.249	1.984 [0.619, 6.359]
Mixed modality access^a^		0.796	0.236	**0.001**	2.216 [1.396, 3.516]
Past-year casino		0.647	0.240	**0.007**	1.910 [1.194, 3.056]
Past-year sports betting		0.468	0.237	**0.049**	1.597 [1.003, 2.542]
Past-year lotteries		0.376	0.221	0.089	1.456 [0.944, 2.426]
Past-year EGMs		0.747	0.350	**0.033**	2.111 [1.064, 4.189]

Statistically significant variables are highlighted in bold

*SE* = standard error; *OR* = odds ratio; *CI* = confidence interval; *EGMs* = electronic gaming machines

^a^Exclusively offline gambling access was used as a reference category

## Discussion

This study aimed at examining gambling behavior in a large sample of young Spanish adults and to examine the risk factors of at-risk/problem gambling. We found that 16.33% of the subjects had gambled in the previous year, and that the prevalence rates of low-risk, moderate-risk, and problem gambling were 3.51%, 2.14% and 0.51%, respectively, in the total sample and 21.51%, 13.08% and 3.10%, respectively, in the past-year gamblers. Several gambling-related characteristics were significantly associated with at-risk/problem gambling, including early onset of gambling, mixed gambling modality vs. exclusively offline, and betting either offline or online in casinos, on sports, and EGMs.

The proportion of participants who engaged in gambling activities online or offline within the previous year was 16.33%, a figure that is lower than the prevalence observed among Spaniards aged between 15 and 24 years from the general population (24% online and 6.5% offline; National Plan on Drugs, 2022). Similarly, this rate was lower than what was reported in a recent meta-analysis among over-18 s worldwide (46.2%; Tran et al., 2024). However, our prevalence is more closely aligned with that observed in young US adults aged 18–25 years, which is the same age range as in our study (22.4%; Noel et al., 2023).

The problem gambling rate of 0.51% in the total sample in our study is lower than the rates reported in previous studies in the young Spanish population (Chóliz et al., 2021; López-Del-Hoyo et al., 2022). However, comparisons for this finding are limited by differences in sample characteristics (e.g., university students vs. the general population) or the instruments assessing problem gambling (e.g., PGSI vs. South Oaks Gambling Screen). From an international perspective, the prevalence of problem gambling in our study is lower than the prevalence of 4.04% estimated in a recent meta-analysis in young adults across European countries (Dellosa & Browne, 2024). The observed differences may be attributed to the age range included in that meta-analysis (young adults aged 18 to 35, while our study included individuals aged 18–25). It is worth noting that a considerable proportion of past-year gamblers in our study exhibited low-risk or moderate-risk problem gambling (21.51% and 13.08% respectively) and could potentially transition to problem gambling in the future. According to prior literature, this risk is particularly pronounced if low- and moderate-risk problem gamblers exhibit additional risk factors, such as being male, impulsivity, or involvement in a greater number of gambling activities (Dowling et al., 2017).

At-risk/problem gambling was associated with several gambling-related characteristics. In line with previous findings (Landreat et al., 2020; Pérez, 2024), we found that early onset of gambling increased the likelihood of at-risk/problem gambling. This underscores the importance of reinforcing gambling control policies to prevent or delay gambling onset during adolescence (e.g., restricting gambling advertising, implement modification of gambling products, limit accessibility and exposure) (Engebø et al., 2021; Planzer et al., 2014) to reduce the risk of adolescents and young adults developing gambling-related problems.

In line with findings from previous studies (Marmet et al., 2021; Papineau et al., 2018), a mixed gambling modality increased the risk of at-risk/problem gambling compared with exclusively offline gambling. Betting online and offline means widespread availability of gambling (e.g., greater opportunities to gamble on different types of activities and contexts, and at times of the day), making this dual modality of gambling potentially more problematic (Díaz & Pérez, 2021; Tomei et al., 2022). This suggests that early detection of vulnerable groups of gamblers needs to target those gambling in both modalities (Marmet et al., 2021).

In addition, we found that having gambled either online or offline in casinos, sports betting, or on EGMs during the previous year increases the risk of being at-risk/problem gambler. The harmful effects of sports betting have been widely demonstrated in the Spanish population, and research links these findings to an interaction between different structural characteristics not found in other gambling modalities, such as watching sports, live in-play betting, fantasy sports gaming, instant technology, and media factors (Lopez-Gonzalez et al., 2019). Sports betting is the preferred online activity among Spanish young adults (National Plan on Drugs, 2022), and there is a proliferation of opportunities to gamble on sports in Spain (Barrera-Algarín & Vázquez-Fernández, 2021). The relationship between having gambled in casinos or on EGMs and problem gambling has also been found by previous studies (Gainsbury et al., 2019; Oksanen et al., 2021). In line with these findings, public policies should consider measures to curb the growth of these types of gambling activities both online (e.g., limit the number of bets allowed per day) and offline (e.g., impose a maximum number of gambling venues per area).

The results of our study should be interpreted considering several limitations. First, the cross-sectional nature of the study prevented us from establishing causal relationships. Second, since problem gambling is more prevalent among men (Allami et al., 2021; Dowling et al., 2017), the power to detect sex differences may have been constrained by the study sample's predominantly female composition (64.16%). Furthermore, in addition to the high percentage of female participants, the young mean age of the total sample (19.47 ± 1.64) considering the range of age included in the study (18–25) indicates the need for caution when generalizing the findings. In addition, the participants were recruited what was considered a “post-pandemic” period, and it is possible that gambling behavior was influenced by the proximity of social restrictions implemented to control the spread of the COVID-19 in the months prior to the recruitment period. This could have impacted the prevalence of problem gambling, as some studies have indicated that gambling activity increased during the pandemic (Andersson et al., 2022), while others found that it remained stable overall (Auer et al., 2020), or declined (Heirene et al., 2021).

## Conclusions

This research contributes to greater knowledge about problem gambling and related variables in the Spanish context and provides insights for policymakers and health professionals that may be useful for preventing gambling problems in young populations. Our study indicates that the prevalence of low-risk and moderate-risk problem gambling is considerable in young adults who had engaged in gambling activities over the past year, with respective rates of 21.51% and 13.08%. Furthermore, early onset of gambling increased the likelihood of at-risk/problem gambling. These findings suggest the need for specific interventions to reduce the likelihood of the onset of gambling in young adults. The implementation of environmental prevention strategies to reduce the availability of gambling opportunities both online and offline (e.g., limitation of gambling marketing or gambling venue locations) may delay gambling onset and mitigate gambling-related harm among young adults, as recent research has suggested (Marionneau et al., 2023; Ukhova et al., 2024).,

Additionally, the implementation of early intervention strategies for those who start gambling at younger ages and who bet in a mixed modality in casino settings, sports betting, and EGMs may prove effective in preventing adverse consequences in emerging adulthood. In this context, university prevention services should consider prioritizing screening for gambling and referral for treatment if necessary. For example, personalized normative feedback has demonstrated efficacy in reducing harm among young adults (Grande-Gosende et al., 2020; Monreal-Bartolomé et al., 2023).

## Supplementary Information

Below is the link to the electronic supplementary material.

Supplementary file1 (DOCX 40 kb)

## Data Availability

Data available from the corresponding author upon reasonable request.
