# Supplementary material for: Risk Factors of At-Risk/Problem Gambling Among Young Adult Spanish Students
Source: J Prev (2022). 2024 Nov 21;46(2):231–44. doi: 10.1007/s10935-024-00814-x (PMC11982148; doi:10.1007/s10935-024-00814-x)
Supplement: Supplementary file 1 — Supplementary file1 (DOCX 40 kb) [file 10935_2024_814_MOESM1_ESM.docx]

**Table S1.**

*Full model from the backward stepwise logistic regression of sociodemographic and gambling-related characteristics for respondents with at-risk/problem gambling*

|  |  | *B* | *SE* | Wald | *df* | *p* | *OR* | 95% CI | |
| --- | --- | --- | --- | --- | --- | --- | --- | --- | --- |
| Step 1 | Male | 0.223 | 0.252 | 0.786 | 1 | 0.375 | 1.250 | 0.763 | 2.047 |
|  | Age at gambling onset | -0.223 | 0.084 | 6.990 | 1 | 0.008 | 0.800 | 0.678 | 0.944 |
|  | Exclusively offline access |  |  | 4.488 | 2 | 0.106 |  |  |  |
|  | Exclusively online access | 0.631 | 0.697 | 0.820 | 1 | 0.365 | 1.880 | 0.480 | 7.373 |
|  | Mixed modality access | 0.702 | 0.331 | 4.488 | 1 | 0.034 | 2.018 | 1.054 | 3.863 |
|  | Non-strategic gambler |  |  | 1.197 | 2 | 0.550 |  |  |  |
|  | Strategic gambler | -0.343 | 0.525 | 0.428 | 1 | 0.513 | 0.709 | 0.253 | 1.985 |
|  | Mixed gambler | -0.496 | 0.469 | 1.119 | 1 | 0.290 | 0.609 | 0.243 | 1.526 |
|  | N gambling activities offline | -0.038 | 0.143 | 0.069 | 1 | 0.793 | 0.963 | 0.727 | 1.276 |
|  | N gambling activities online | -0.040 | 0.133 | 0.090 | 1 | 0.765 | 0.961 | 0.740 | 1.248 |
|  | Past-year casino | 0.808 | 0.343 | 5.562 | 1 | 0.018 | 2.243 | 1.146 | 4.391 |
|  | Past-year sports betting | 0.537 | 0.289 | 3.448 | 1 | 0.063 | 1.711 | 0.971 | 3.016 |
|  | Past-year lotteries | 0.453 | 0.292 | 2.403 | 1 | 0.121 | 1.573 | 0.887 | 2.788 |
|  | Past-year scratch cards | 0.278 | 0.303 | 0.840 | 1 | 0.359 | 1.320 | 0.729 | 2.390 |
|  | Past-year EGMs | 0.788 | 0.422 | 3.483 | 1 | 0.062 | 2.199 | 0.961 | 5.030 |
|  | Past-year trading | 0.340 | 0.345 | 0.969 | 1 | 0.325 | 1.404 | 0.714 | 2.761 |
|  | Past-year private bets | 0.208 | 0.297 | 0.489 | 1 | 0.484 | 1.231 | 0.687 | 2.205 |
|  | Constant | 2.264 | 1.562 | 2.102 | 1 | 0.147 | 9.621 |  |  |
| Step 2 | Male | 0.223 | 0.252 | 0.783 | 1 | 0.376 | 1.250 | 0.763 | 2.047 |
|  | Age at gambling onset | -0.223 | 0.084 | 7.020 | 1 | 0.008 | 0.800 | 0.678 | 0.944 |
|  | Exclusively offline access |  |  | 4.775 | 2 | 0.092 |  |  |  |
|  | Exclusively online access | 0.684 | 0.667 | 1.054 | 1 | 0.305 | 1.983 | 0.537 | 7.323 |
|  | Mixed modality access | 0.715 | 0.328 | 4.757 | 1 | 0.029 | 2.045 | 1.075 | 3.888 |
|  | Non-strategic gambler |  |  | 1.268 | 2 | 0.531 |  |  |  |
|  | Strategic gambler | -0.313 | 0.512 | 0.375 | 1 | 0.541 | 0.731 | 0.268 | 1.994 |
|  | Mixed gambler | -0.495 | 0.468 | 1.116 | 1 | 0.291 | 0.610 | 0.243 | 1.527 |
|  | N gambling activities online | -0.040 | 0.134 | 0.090 | 1 | 0.764 | 0.961 | 0.739 | 1.249 |
|  | Past-year casino | 0.764 | 0.299 | 6.541 | 1 | 0.011 | 2.148 | 1.196 | 3.859 |
|  | Past-year sports betting | 0.509 | 0.268 | 3.606 | 1 | 0.058 | 1.663 | 0.984 | 2.811 |
|  | Past-year lotteries | 0.423 | 0.269 | 2.469 | 1 | 0.116 | 1.527 | 0.901 | 2.588 |
|  | Past-year scratch cards | 0.240 | 0.267 | 0.809 | 1 | 0.368 | 1.271 | 0.754 | 2.145 |
|  | Past-year EGMs | 0.731 | 0.362 | 4.072 | 1 | 0.044 | 2.078 | 1.021 | 4.227 |
|  | Past-year trading | 0.325 | 0.341 | 0.913 | 1 | 0.339 | 1.385 | 0.710 | 2.699 |
|  | Past-year private bets | 0.160 | 0.234 | 0.467 | 1 | 0.495 | 1.173 | 0.742 | 1.856 |
|  | Constant | 2.234 | 1.554 | 2.065 | 1 | 0.151 | 9.333 |  |  |
| Step 3 | Male | 0.221 | 0.251 | 0.774 | 1 | 0.379 | 1.248 | 0.762 | 2.042 |
|  | Age at gambling onset | -0.219 | 0.083 | 6.992 | 1 | 0.008 | 0.803 | 0.682 | 0.945 |
|  | Exclusively offline access |  |  | 6.345 | 2 | 0.042 |  |  |  |
|  | Exclusively online access | 0.612 | 0.622 | 0.969 | 1 | 0.325 | 1.844 | 0.545 | 6.239 |
|  | Mixed modality access | 0.657 | 0.263 | 6.230 | 1 | 0.013 | 1.928 | 1.151 | 3.229 |
|  | Non-strategic gambler |  |  | 1.229 | 2 | 0.541 |  |  |  |
|  | Strategic gambler | -0.293 | 0.507 | 0.334 | 1 | 0.563 | 0.746 | 0.276 | 2.016 |
|  | Mixed gambler | -0.480 | 0.465 | 1.064 | 1 | 0.302 | 0.619 | 0.249 | 1.540 |
|  | Past-year casino | 0.748 | 0.293 | 6.500 | 1 | 0.011 | 2.112 | 1.189 | 3.754 |
|  | Past-year sports betting | 0.498 | 0.265 | 3.523 | 1 | 0.061 | 1.646 | 0.978 | 2.770 |
|  | Past-year lotteries | 0.424 | 0.269 | 2.476 | 1 | 0.116 | 1.528 | 0.901 | 2.591 |
|  | Past-year scratch cards | 0.235 | 0.266 | 0.779 | 1 | 0.377 | 1.265 | 0.751 | 2.133 |
|  | Past-year EGMs | 0.710 | 0.356 | 3.982 | 1 | 0.046 | 2.034 | 1.013 | 4.084 |
|  | Past-year trading | 0.288 | 0.316 | 0.827 | 1 | 0.363 | 1.333 | 0.717 | 2.478 |
|  | Past-year private bets | 0.152 | 0.233 | 0.428 | 1 | 0.513 | 1.164 | 0.738 | 1.837 |
|  | Constant | 2.172 | 1.536 | 2.001 | 1 | 0.157 | 8.779 |  |  |
| Step 4 | Male | 0.211 | 0.249 | 0.712 | 1 | 0.399 | 1.234 | 0.757 | 2.012 |
|  | Age at gambling onset | -0.216 | 0.083 | 6.736 | 1 | 0.009 | 0.806 | 0.685 | 0.949 |
|  | Exclusively offline access |  |  | 6.125 | 2 | 0.047 |  |  |  |
|  | Exclusively online access | 0.577 | 0.611 | 0.891 | 1 | 0.345 | 1.780 | 0.538 | 5.893 |
|  | Mixed modality access | 0.643 | 0.262 | 6.034 | 1 | 0.014 | 1.902 | 1.139 | 3.178 |
|  | Past-year casino | 0.599 | 0.242 | 6.109 | 1 | 0.013 | 1.820 | 1.132 | 2.925 |
|  | Past-year sports betting | 0.416 | 0.248 | 2.823 | 1 | 0.093 | 1.516 | 0.933 | 2.463 |
|  | Past-year lotteries | 0.380 | 0.240 | 2.505 | 1 | 0.114 | 1.462 | 0.913 | 2.341 |
|  | Past-year scratch cards | 0.161 | 0.247 | 0.427 | 1 | 0.513 | 1.175 | 0.725 | 1.905 |
|  | Past-year EGMs | 0.714 | 0.354 | 4.065 | 1 | 0.044 | 2.041 | 1.020 | 4.085 |
|  | Past-year trading | 0.270 | 0.314 | 0.740 | 1 | 0.390 | 1.310 | 0.708 | 2.423 |
|  | Past-year private bets | 0.137 | 0.230 | 0.353 | 1 | 0.553 | 1.146 | 0.730 | 1.800 |
|  | Constant | 1.942 | 1.519 | 1.635 | 1 | 0.201 | 6.975 |  |  |
| Step 5 | Male | 0.222 | 0.248 | 0.801 | 1 | 0.371 | 1.249 | 0.768 | 2.032 |
|  | Age at gambling onset | -0.217 | 0.083 | 6.820 | 1 | 0.009 | 0.805 | 0.683 | 0.947 |
|  | Exclusively offline access |  |  | 6.748 | 2 | 0.034 |  |  |  |
|  | Exclusively online access | 0.557 | 0.610 | 0.833 | 1 | 0.362 | 1.745 | 0.528 | 5.773 |
|  | Mixed modality access | 0.669 | 0.258 | 6.709 | 1 | 0.010 | 1.952 | 1.177 | 3.239 |
|  | Past-year casino | 0.600 | 0.242 | 6.136 | 1 | 0.013 | 1.822 | 1.133 | 2.928 |
|  | Past-year sports betting | 0.412 | 0.247 | 2.769 | 1 | 0.096 | 1.510 | 0.929 | 2.452 |
|  | Past-year lotteries | 0.385 | 0.240 | 2.570 | 1 | 0.109 | 1.470 | 0.918 | 2.353 |
|  | Past-year scratch cards | 0.162 | 0.247 | 0.434 | 1 | 0.510 | 1.176 | 0.725 | 1.908 |
|  | Past-year EGMs | 0.732 | 0.352 | 4.308 | 1 | 0.038 | 2.078 | 1.042 | 4.147 |
|  | Past-year trading | 0.278 | 0.313 | 0.787 | 1 | 0.375 | 1.320 | 0.714 | 2.441 |
|  | Constant | 2.001 | 1.521 | 1.730 | 1 | 0.188 | 7.395 |  |  |
| Step 6 | Male | 0.206 | 0.247 | 0.699 | 1 | 0.403 | 1.229 | 0.758 | 1.995 |
|  | Age at gambling onset | -0.220 | 0.083 | 6.991 | 1 | 0.008 | 0.803 | 0.682 | 0.945 |
|  | Exclusively offline access |  |  | 7.276 | 2 | 0.026 |  |  |  |
|  | Exclusively online access | 0.538 | 0.610 | 0.780 | 1 | 0.377 | 1.713 | 0.519 | 5.657 |
|  | Mixed modality access | 0.691 | 0.256 | 7.264 | 1 | 0.007 | 1.995 | 1.207 | 3.297 |
|  | Past-year casino | 0.609 | 0.242 | 6.335 | 1 | 0.012 | 1.838 | 1.144 | 2.952 |
|  | Past-year sports betting | 0.412 | 0.247 | 2.773 | 1 | 0.096 | 1.510 | 0.930 | 2.453 |
|  | Past-year lotteries | 0.435 | 0.228 | 3.636 | 1 | 0.057 | 1.545 | 0.988 | 2.415 |
|  | Past-year EGMs | 0.745 | 0.351 | 4.494 | 1 | 0.034 | 2.107 | 1.058 | 4.195 |
|  | Past-year trading | 0.267 | 0.313 | 0.729 | 1 | 0.393 | 1.306 | 0.707 | 2.413 |
|  | Constant | 2.071 | 1.516 | 1.867 | 1 | 0.172 | 7.933 |  |  |
| Step 7 | Age at gambling onset | -0.230 | 0.083 | 7.733 | 1 | 0.005 | 0.795 | 0.676 | 0.934 |
|  | Exclusively offline access |  |  | 7.310 | 2 | 0.026 |  |  |  |
|  | Exclusively online access | 0.560 | 0.609 | 0.845 | 1 | 0.358 | 1.751 | 0.530 | 5.780 |
|  | Mixed modality access | 0.691 | 0.256 | 7.287 | 1 | 0.007 | 1.995 | 1.208 | 3.295 |
|  | Past-year casino | 0.617 | 0.241 | 6.526 | 1 | 0.011 | 1.853 | 1.154 | 2.974 |
|  | Past-year sports betting | 0.470 | 0.237 | 3.926 | 1 | 0.048 | 1.601 | 1.005 | 2.549 |
|  | Past-year lotteries | 0.394 | 0.222 | 3.147 | 1 | 0.076 | 1.483 | 0.960 | 2.291 |
|  | Past-year EGMs | 0.745 | 0.351 | 4.517 | 1 | 0.034 | 2.107 | 1.060 | 4.188 |
|  | Past-year trading | 0.321 | 0.306 | 1.100 | 1 | 0.294 | 1.379 | 0.756 | 2.513 |
|  | Constant | 2.345 | 1.485 | 2.496 | 1 | 0.114 | 10.437 |  |  |
| Step 8 | Age at gambling onset | -0.236 | 0.082 | 8.179 | 1 | 0.004 | 0.790 | 0.672 | 0.929 |
|  | Exclusively offline access |  |  | 11.536 | 2 | 0.003 |  |  |  |
|  | Exclusively online access | 0.685 | 0.594 | 1.328 | 1 | 0.249 | 1.984 | 0.619 | 6.359 |
|  | Mixed modality access | 0.796 | 0.236 | 11.399 | 1 | 0.001 | 2.216 | 1.396 | 3.516 |
|  | Past-year casino | 0.647 | 0.240 | 7.291 | 1 | 0.007 | 1.910 | 1.194 | 3.056 |
|  | Past-year sports betting | 0.468 | 0.237 | 3.893 | 1 | 0.049 | 1.597 | 1.003 | 2.542 |
|  | Past-year lotteries | 0.376 | 0.221 | 2.890 | 1 | 0.089 | 1.456 | 0.944 | 2.246 |
|  | Past-year EGMs | 0.747 | 0.350 | 4.563 | 1 | 0.033 | 2.111 | 1.064 | 4.189 |
|  | Constant | 2.445 | 1.481 | 2.725 | 1 | 0.099 | 11.527 |  |  |
| *Note.* SE = standard error; df = degrees of freedom; OR = odds ratio; CI = confidence interval; EGMs = electronic gaming machines. | | | | | | | | | |
